# Perception, environmental determinants, and health complications of excess weight in India: a mixed methods approach

**DOI:** 10.1038/s41598-023-31016-w

**Published:** 2023-04-11

**Authors:** Somdutta Barua, Nandita Saikia

**Affiliations:** 1 Jawaharlal Nehru University, New Delhi, India; 2 National Institute of Urban Affairs, New Delhi, India; 3 Department of Public Health and Mortality Studies, International Institute for Population Sciences, Mumbai, India

**Keywords:** Nutrition disorders, Environmental social sciences, Environmental impact, Psychology and behaviour, Socioeconomic scenarios

## Abstract

In light of the high and rising prevalence of obesity, we studied females and males aged 25–54 years with excess weight in the Kolkata metropolitan region, one of India's main cities, to understand the perception, environmental determinants and health complications of obesity. We resorted to primary fieldwork. The close-ended quantitative survey questionnaire was designed to capture the perception and health complications of the sampled population, while a semi-structured interview guide comprising open-ended questions was prepared to capture the target population’s in-depth views. Following the WHO guidelines on BMI and WC cut-off for Asian adults, the sampled population comprised females and males aged 25–54 with a waist circumference of 80 cm and 90 cm or higher, respectively, and a BMI of 25 or higher in the Kolkata metropolitan area. Using a concurrent mixed methods approach, we collected and analysed quantitative and qualitative data separately using descriptive statistics and inductive coding before combining them. In the study area, we completed 120 surveys and 18 in-depth interviews. Some environmental factors promoting obesity were the lack of access to healthy, fresh foods, lack of health awareness programmes, advertisements, and weather conditions in Kolkata. Interview participants also extended their concerns about food adulteration and the food industry. Participants confirmed that obesity could increase the risk of developing diabetes, hypertension, cholesterol and heart disease. Further, participants felt squatting was challenging for them. Hypertension was the most common existing health complication found among the study participants. Participants suggested raising awareness, promoting and making access to healthy food and wellness programs more accessible, and regulating fast foods and sugary beverages at institutional, community and social/public policy levels to prevent obesity. Health education and better policies are required to combat obesity and related complications.

## Introduction

According to World Health Organization (WHO), two-thirds of the global burden of diseases are chronic non-communicable diseases, primarily associated with people’s diet^1^, and high energy density foods are the primary agent conditioned by other environmental conditions that result in obesity^2^. Globally, obesity prevalence increased threefold between 1975 and 2016, with 15% of women and 11% of men, i.e. estimated to be 650 million or more individuals with obesity^3^. Since many adults have excess weight in several countries, the WHO consultation report has accentuated that obesity needs to be addressed and understood as a population problem, not an individual one^4^.

It has been estimated that human genes can be accountable for around 40–70% of having obesity. Even though human genetic build-up plays a significant role, influencing the risk of obesity becomes much more robust when it interacts with environmental factors and lifestyle^5^. Understanding the physical environment is vital in determining the risk of obesity. Environmental factors include food marketing and advertising, knowledge regarding exercise and nutrition, availability of resources, local walkability and crime, etc., making the role of the environment multifaceted and complex^6^. Obesogenic neighbourhoods built by ‘food deserts’ make access to healthy and fresh foods challenging. Further, too many fast-food chains contribute to such an environment, selling unhealthy meals with low nutrition and high fat at low prices^7^. Living in such an environment encourages people to make poor food choices and to become less active while making it challenging to maintain a healthy body weight. Therefore, at the neighbourhood level, fast food consumption increases alongside a degraded home diet while promoting the possibility of nutritional morbidities like diabetes and obesity. This is how our environment unconsciously or consciously encourages people to make wrong choices^6^.

Obesity can affect overall health, i.e. physical, mental/psychological, social and cultural, emotional and spiritual health^8^. The long list of associated complications of obesity includes type 2 diabetes^7,9–11^, hypertension, heart problems, stroke, osteoarthritis, gallbladder disease^7,9^, dyslipidaemia, sleep apnea, respiratory problems, polycystic ovarian syndrome^7^, cancers^7,11^, pregnancy complications and depression^7,9,12^. People with excess weight have twice the higher chances of becoming the victim of heart failure than non-obese individuals, and 85 per cent or higher of type 2 diabetes patients are overweight. Individuals with obesity are at higher risk of developing and dying from cardiovascular disease, diabetes, and cancer than normal-weighted subjects. Besides, osteoarthritis risk increases by 9 to 13 per cent with a gain of every two pounds^9^. Even individuals with Body Mass Index (BMI) over 25 exhibited a higher risk of contracting Covid-19^13^, with a 50 per cent increased risk of mortality^14^.

Alongside undernutrition, India is now facing another malnutrition problem, i.e. obesity, with around 20 per cent of the population aged 15–49 years with a BMI of 25 or higher^15^. In 2014, 3.7% of males and 5.3% of females worldwide, who ranked fifth and third, respectively, had excess weight in India, according to the NCD Risk Factor Collaboration research study. The percentages increased from 0.8% for females and 0.4% for males, moving the percentage from 19th place in just three and a half decades^16^. Increasing prevalence can increase the medical burden and decrease labour productivity^17^, which can be challenging in low- and middle-income countries with poorly equipped health systems^5^, lack of sub-optimal services and unqualified carers^18,19^. Despite recent reforms, India’s health system remains challenging because of the low financial investment and increasing non-communicable diseases^13^. India's health system is yet to be well-equipped to deal with non-communicable diseases^20^. Historically, India has had poor financial health coverage for most of the population. Partly due to the low government investment in health, out-of-pocket spending accounted for around 62.6 per cent of total health expenditures in 2015, thrice the global average and one of the world’s highest^18^. Due to the poor government facilities, people avail the private carers^18^. On the other hand, a community-based study in Delhi found that when the government offered quality healthcare services, people who previously went to private healthcare providers opted for community clinics^19^. Even with the insurance plan, several services and treatments related to obesity may not be covered^6^. India has well-drafted, articulated policies and programmes, which are only partially implemented. On the other hand, many people may forego medical care due to the higher costs of stroke, coronary heart disease, diabetes, and other diseases^12^.

In India, the urban population have a higher prevalence of obesity than the rural population^21^. We have chosen Kolkata metropolitan area as the study area since it is the largest urban agglomeration in Eastern India and the oldest metropolis in India^22^. It ranked 7th out of 640 districts, the highest among the five major urban cities in India, with around 43 per cent of the male and 41 per cent of the female population aged 15–49 years with a BMI of 25 or higher^15^. In light of the high and rising prevalence rate in Kolkata, it was worthwhile to study perceptions, environmental determinants, and health complications of excess weight, which could help to design necessary interventions and programs for public wellbeing. This study found the participatory/advocacy philosophical worldview appropriate to deal with the research problem. Furthermore, using mixed methods, no previous studies have explored the perceptions, environmental determinants, and health complications of excess weight among adults with excess weight in the Kolkata metropolitan area. The present study's novelty is that it is based on the recently collected field data, qualitative and quantitative data. The principal investigator (PI) measured each participant's height, weight, and waist circumference.

## Methods

### Research design

**Figure 1:**
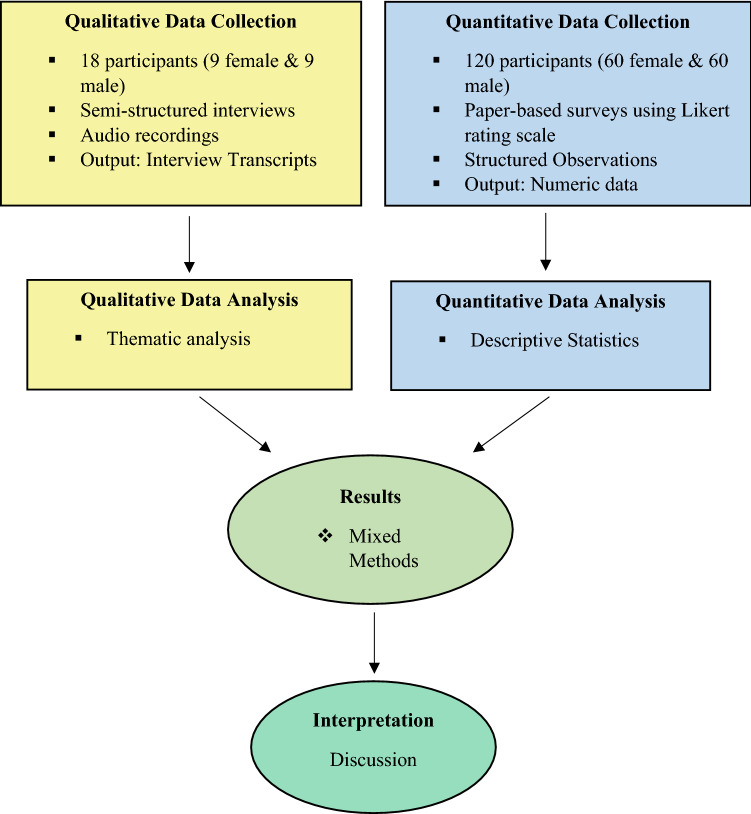
Diagram of concurrent mixed methods design (Forman 2019).

We opted for a concurrent mixed methods design where we separately collected data between November 2019 and October 2020, analysed the quantitative (closed-ended) and qualitative (open-ended) data and then integrated them. We gave equal weight to quantitative and qualitative data^23^. A drawing of the convergent mixed methods design has been presented in Fig. 1^24^. To develop the field instruments and framework for this study, we considered the Social-Ecological Model and the Health Belief Model.

### Sampling, study population and data collection procedure

Purposive and snowball nonprobability sampling procedures were adopted for this study's quantitative and qualitative aspects. In the Asian population, the body fat percentage is 3–5 per cent higher than that of European descent^17^. Hence, the Consensus Guidelines^25^ and WHO^26^ suggested different BMI guidelines for Asian adults and labelled obesity at a BMI of 25 or higher. Similarly, the at-risk waist circumference (WC) cut-off is 90 cm in males and 80 cm in females due to higher odds for cardiovascular risk factors at and above the same range. Following the same guidelines, we opted for purposive sampling because we aimed to examine females and males aged 25–54 years with a BMI of 25 or higher and a waist circumference of 80 cm or 90 cm, respectively, who had lived in the Kolkata metropolitan region for the prior six months. We took this age group and those with excess weight for several reasons. First, it has been mentioned in the literature^12^ that people tend to gain weight as they get older, especially in their middle years. Second, this group was selected because it represented the years when an individual was most productive. Careers are built, marriages occur, and children are reared during this time. Hence, it is essential to prioritise a healthy lifestyle and establish preventative health habits for oneself and their family. Last but not least, previous research has shown that people with excess weight are more likely to experience clinical and sociological complications. Furthermore, we decided to do snowball sampling because there was no data to single out the intended study population, and we did not choose a door-to-door or household survey due to the sensitivity of this topic and the potential stigma attached to bigger bodies. PI reached out to the participants via referral, phone, or social media, and the rest PI recruited with the help of other research participants. PI pre-informed the interviewees that interviews would be audio recorded, and PI took prior consent with a signature on the consent form. PI measured height, weight and waist circumference for every participant using a standard long tape, a weighing machine and a waist measuring tape, respectively. PI gave out the quantitative questionnaires to the participants or administered them during the fill-up, depending on the respondents' preference. PI assigned a pseudonym for the interview participants. Saturation was reached with the completion of eighteen interviews. The investigator tried to be as harmonic, unbiased, respectful, and non-judgmental during the interviews and surveys^27^. Jawaharlal Nehru University's Institutional Ethics Review Board (IERB-JNU) granted pre-approval for data collection. The final results have been reported truthfully to uphold the study's ethics^27^. The PI conducted the entire survey and the interview procedure.

### Instruments used for data collection

The closed-ended quantitative survey questionnaire provided a numeric portrayal of the perceptions of the sampled population^23^. There were 28 items to answer that used a five-point level of agreement Likert scale, whose Cronbach's alpha was 0.76, 4 items that used the five-point level of importance on the Likert scale, whose Cronbach's alpha was 0.73 and another 5 items that used the two-point dichotomous Likert scales, whose Cronbach's alpha was 0.37. With the assistance of three native Bengali speakers, the English quantitative survey questionnaire was translated into the regional language, Bengali. The survey took about ten minutes to complete.

To gain a more in-depth insight into the target population's perception, we created a semi-structured interview guide with open-ended questions aligned with the quantitative survey questionnaire and research problem. PI audio-recorded the discussions while conducting face-to-face interviews. We asked six semi-structured questions in total. PI administered the interviews with voluntarily agreed-upon participants in their native languages (Bengali, English, and Hindi), designed to allow the interviewees to speak their minds freely. An interview lasted an average of 20 to 25 min to complete.

### Field experiences

Between November 2019 and October 2020, the PI visited the field. The covid-19 pandemic, weight-related prejudice, and lack of interest, time, and motivation made it challenging to conduct surveys and interviews. The final results included 120 quantitative surveys and 18 in-depth interviews.

### Analysis of data

#### Univariate analysis

We used descriptive statistics to present the percentage and frequency of demographics and responses of the sampled individuals with the help of Stata 14.0. We estimated the BMI by inputting each participant's height (cm) and weight (kg) into the National Institutes of Health (NIH) website.

#### Coding of themes

We followed Creswell's^23^ six-step approach for analysing qualitative data: a) Transcribing the audio recording and writing up the field notes b) Re-reading the data to evaluate the overall meaning and credibility of the transcripts; c) Writing a code for the relevant sections that is an abbreviation of the given topic; and d) These codes are then developed to construct broad themes as a representation of the study findings e) Making connections between narrative and the themes; f) documenting the qualitative data alongside with the literature. The lead investigator transcribed the interviews, which was laborious and took approximately 1–2 h per interview. In this study, we focused on three prominent themes.

#### Merging results of two datasets

We used 'joint displays' to combine, compare, and exhibit the results by juxtaposing (side-by-side) the qualitative and quantitative data^23^.

### Human and animal ethics

The Institutional Ethics Review Board of Jawaharlal Nehru University (IERB-JNU) gave the pre-approval for collecting data and approved the study protocol. Furthermore, informed consent was obtained from all subjects and we obtained their approval via a signature on a consent form. The authors confirm that all methods have been carried out in accordance with the relevant guidelines and regulations. All data are kept anonymous and confidential. This research study did not involve the use of biological or human tissue samples.

## Results

### Descriptive statistics of the survey participants

The study enlisted the participation of 120 persons, 60 of whom were females and 60 of whom were males. The descriptive statistics of study participants aged 25 to 54 years, with an average age of 39 years, are shown in Table 1^24^. The participants in the study were mainly between the ages of 25 and 34. Half of the respondents had obtained their diplomas or had completed their college education. The average monthly family income of the participants was between 40,001 and 80,000 INR, and they were generally employed, with a household size of around four people. Most of those who took the survey were Hindu believers, from the General or Other Backward Classes (OBC) social group and spoke Bengali as their native language. The majority of the people who took part were married and had children. In addition, more participants had a BMI of 30 or higher, a waist circumference of 102 cm or higher, and were nonsmokers. The average BMI of the study subjects was 31.28, with a waist circumference of 106.44 cm. In addition, 45.83 per cent of those who took the survey never consumed alcohol, and one-fifth of them had hypothyroidism.

**Table 1:** Descriptive statistics of the survey participants, Kolkata, 2019–20.

Background characteristics	Frequency (n)	Percentage (%)
Age (in years)		
25–34	44	36.67
35–44	33	27.5
45–54	43	35.83
Education level		
Up to Secondary	15	12.5
Up to Graduation	65	54.17
P.G. or Higher	40	33.33
Monthly family income		
Up to 40,000	25	20.83
40,001 to 80,000	39	32.5
Above 80,000	37	30.83
Don't Know/Prefer not to answer	19	15.83
Employment status		
Not employed (Unpaid)	27	22.50
Employed	93	77.50
Household members		
Up to five members	112	93.33
More than five members	8	6.67
Religious beliefs		
Hinduism	85	70.83
Islam	5	4.17
Other	30	25
Social group		
SC/ST	8	6.67
General/OBC	105	87.5
Can’t Say/Don’t Know	7	5.83
Mother tongue		
Bengali	109	90.83
Others	11	9.17
Marital status		
Unmarried	31	25.83
Married	89	74.17
Have children		
Yes	79	65.83
No	41	34.17
Body Mass Index		
25 up to below 30	59	49.17
30 or higher	61	50.83
Waist circumference		
Up to 102 cm	51	42.5
More than 102 cm	69	57.5
Smoking		
Regular	31	25.83
Occasional	14	11.67
Former	8	6.67
Never	67	55.83
Alcohol consumption		
Regular	10	8.33
Occasional	53	44.17
Former	2	1.67
Never	55	45.83
Health problems^		
PCOS*	8	13.33
Thyroid	25	20.83
Arthritis	6	5.00
Acidity/Gas	4	3.33
Mental health condition	4	3.33
Hypertension	45	37.50
High blood sugar	20	16.67
Sleep apnea	26	21.67
Asthma	13	10.83
Cholesterol	24	20
Other health issues	12	10
No health problem	35	29.17
Total	120	

^Had suffered or presently living with the specific health problems and/or under the medication.

*Among 60 female participants.

Source: Primary Surv﻿ey.

### Descriptive statistics of the in-depth interviewees

Nine females and nine males, 18 individuals, volunteered to participate in the interview (Table 2)^24^. The interviewers' mean age was also 39, ranging from 27 to 52 years. Most participants had graduated, were employed, had a monthly family income of more than 80,000 INR, and generally lived in four-person households on average. Hinduism was the most common religious belief among the sampled participants, who were mainly from the 'General' social category and spoke Bengali as their first language. Most of those interviewed were married people with children. Further, most sample interviewees had a waist circumference of more than 102 cm and a BMI of over 30. The average BMI of the participants was 32.87, with a waist circumference of 109.28 cm. Furthermore, nearly half of the participants stated that they had never smoked, occasionally drank alcohol, had at least one health concern, or were on medication.

**Table 2:** Descriptive statistics of the interviewees, Kolkata, 2019–2﻿0.

Partcicipant no	Age (in years)	Sex	Education level	Occupation	Monthly family income (INR)	Household members	Religious belief	Caste	Mother tongue	Marital status	Have children	BMI	WC (in cm)	Smoking	Alcohol consumption	Known complication/under medication
1	27	Female	Post Graduate	Service	Above 80,000	4	Hinduism	General	Bengali	Unmarried	No	33.5	117.5	Never	Occasional	N/A
2	31	Female	Post Graduate	Service	Above 80,000	4	Hinduism	General	Bengali	Married	Yes	30.3	105	Occasional	Occasional	N/A
3	33	Female	Graduate	Business	Above 80,000	4	Hinduism	General	Bengali	Married	No	37.3	123	Never	Former	PCOS; Hypertension; Cholesterol
4	37	Female	Graduate	Business	Above 80,000	3	Hinduism	OBC	Hindi/Bhojpuri	Married	Yes	26.6	98	Never	Occasional	Thyroid
5	38	Female	9th Standard	Homemaker	Don't Know	4	Hinduism	Can't Say/Don't Know	Bengali	Married	Yes	26.5	93	Never	Never	N/A
6	40	Female	Post Graduate	Service	40,001–80,000	4	Omnism	General	Bengali	Separated	Yes	35.9	102	Never	Never	N/A
7	45	Female	Graduate	Homemaker	Above 80,000	3	Hinduism	General	Bengali	Married	Yes	36.3	112	Never	Never	PCOS; Thyroid; Type 2 Diabetes; Hypertension
8	46	Female	Graduate	Freelance Writer	Up to 40,000	3	Hinduism	General	Bengali	Married	Yes	31.6	99.5	Occasional	Occasional	Migraine
9	51	Female	Graduate	Freelance Researcher	Above 80,000	4	Islam	No Caste	Bengali	Married	Yes	41.5	119	Occasional	Occasional	Type 2 Diabetes; Hypertension
10	26	Male	Post Graduate	Private Tutor	40,001–80,000	4	Humanism	ST	Bengali	Unmarried	No	33.3	111	Occasional	Occasional	N/A
11	28	Male	Post Graduate	Research Scholar	Above 80,000	4	Hinduism	General	Bengali	Unmarried	No	33.2	108	Occasional	Occasional	N/A
12	30	Male	Post Graduate	Private Tutor	40,001–80,000	3	Non-believer	General	Bengali	Unmarried	No	27.1	96	Occasional	Occasional	Asthma
13	37	Male	Higher Secondary	Service	Up to 40,000	2	Non-believer	General	Bengali	Unmarried	No	31.6	104	Never	Occasional	Gas
14	41	Male	Graduate	Business	40,001–80,000	5	Hinduism	General	Bengali	Married	Yes	31.4	111	Regular	Occasional	Type 2 Diabetes; Hypertension
15	43	Male	Graduate	Service & Business	Up to 40,000	8	Hinduism	General	Bengali	Married	Yes	34.3	121	Never	Never	High Blood Sugar
16	47	Male	Graduate	Service	40,001–80,000	2	Hinduism	General	Bengali	Widowed	Yes	30.8	106	Regular	Former	N/A
17	50	Male	Higher Secondary	Business	40,001–80,000	4	Humanism	General	Bengali	Married	Yes	37.5	127	Never	Regular	Hypertension; Cholesterol
18	52	Male	Graduate	Service/Author	Above 80,000	2	Non-believer	General	Bengali	Married	Yes	32.9	114	Former	Occasional	Type 2 Diabetes; Hypertension

Source: Primary Surv﻿ey.

### Themes

Three broad themes emerged from the qualitative data: 1) environmental determinants, 2) health complications, 3) preventing obesity.

### Environmental determinants

A side-by-side comparison of quantitative and qualitative findings on environmental determinants is shown in Table 3. The table is a *side-by-side* display with three sections: The first column displays the type of integration for each domain and the relationship between the two types of data. The quantitative results appear in the second column, while the qualitative results appear in the third. By confirmation, we signify that two types of evidence agree; by expansion, we mean that understanding is expanding. On the other hand, the unstructured observations are generally derived from the PI's field visits and while collecting the surveys.

See Table 3.

**Table 3:** Juxtaposed findings of quantitative and qualitative investigation on environmental determinants. Source: Primary Survey.

Type of integration/domain	Quantitative survey findings	Qualitative interview findings
*Confirmation* Fast foods Concern about the accessibility of healthy produce Food advertising Lack of educational programs Safe neighbourhood *Expansion* Food adulteration Packaged foods Profit-making industries (food, medical, and weight loss)	Participants agreed that the easy access to fast food (39.17% agree; 53.33% strongly agree), lack of accessibility to fresh produce and healthy food at a reasonable price (33.33% agree, 16.24% strongly agree), high-calorie food advertising (36.67% agree, 19.17% strongly agree) increased the risk of obesity.	*"It is not that fast food was not here before. Oily foods were there. But the sort of fast food like burgers, fries which are upcoming. Sugar intake, cafes, frappes, these are quite elementary."* – Arpita *"Pizza is of Italian climate, not the climate of India. But because India is basically a literate illiterate country, you can sell anything here. And people would take to some kind of a fashion just to show themselves better in life or flourishing in life, upwardly mobile in life."* – Sumit *"Now, there is another paradox that is coming up of organic food and healthy food, which are highly expensive. And then, who can really afford to have organic food? That affordability part has to be dealt with also."* – David Ashley saw several billboards which were advertising food, particularly chips. She discussed, *"Food-related TV ads are found – KFC burgers available at these places at this lesser price. It is fully provocative. Now you sit at home and order on Swiggy, Zomato – pizzas, burgers which I think is destroying the habit of Kolkata."* *"They give discounts as well. You tend to order food after seeing discounts." –* Tina
	Participants frequently disagreed (37.61% disagree & 17.09% strongly disagree) that any health awareness programmes in their community focused on obesity. However, they agreed (56.67% agree; 16.67% strongly agree) that the presence of parks, open spaces, walkability, and wellness facilities in their community helped to prevent obesity. More than half of the survey respondents disagreed that littering on the streets (59.66% disagree), stray dogs (35.29% disagree & 33.61% strongly disagree), poor lighting, unsafe neighbourhood and fast traffic (23.73% disagree & 31.36% strongly disagree), and lack of public transport (20% disagree & 46.67% strongly disagree) discouraged them from participating in physical activities	*"The food industry – how safe it is? How much healthy? That's another issue which we do not see. And along with that, how physical health issues are linked to that is also being overlooked. There is no discussion. Its more to do with you are obese, so you are yourself responsible. But what are my options? What is the link between food and weight? How is it being produced? The meat industry – how many types of antibiotics are being used, which is going inside my body? And in the future, if I fall sick, how the antibiotics are not working because of those things – I think about these links. Is it being talked enough? And that is a big problem."* – Naomi
	*Unstructured observations* All kinds of ready-made food Swiggy and Zomato are the leading food delivery apps Advertisements for different foods, illnesses and weight loss methods are found on various platforms (phones, newspapers, TV, billboards, etc.)	*"The market near my house is very good. You get fish there, vegetables, fruits. But there is an adulteration issue in our city. I don't know how to avoid it. Maybe I am buying the stuff, but I doubt whether it is healthy or not."* – Charlie Nancy used phrases like '*not in pure form', 'adulterated'* and *'vegetables are almost hybrid'*. She added, *"Everyone has gastric issues. It's because of excess fertilisation and excess use of urea"*
		Naomi felt, *"There is a whole lot of package food in India, which I think is a problem."*, additionally David stated, *"but at the same time, there is an entire industry of gym – which is also upcoming at the same time"* *"The society is profit-oriented, does not care about my health."* – Charlie

### Health complications

Table 4 is a *joint display* combining quantitative and qualitative findings on health complications. The green colours indicate agreement, and the blues imply disagreement as per the maximum study participants’ responses to the associated health complications. Furthermore, the grey colour specifies the inconclusive answers, while red demonstrates the existing health complications among the study respondents.

**Table 4:**
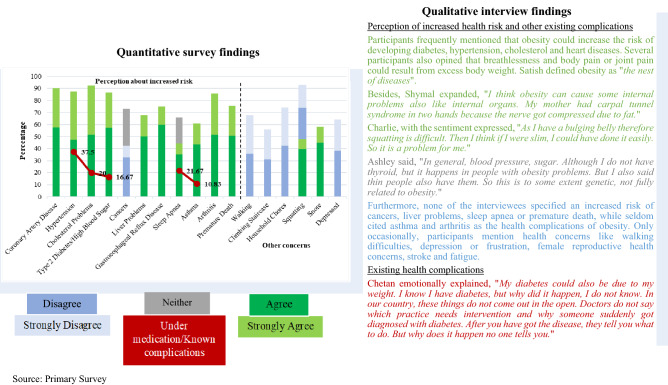
Juxtaposed findings of quantitative and qualitative investigation on health complications.

### Preventing obesity

Table 5 is the final side-by-side display combining quantitative and qualitative findings on preventing obesity. The green colours indicate the agreement, and the grey colours pinpoint the strategies to prevent obesity as per the maximum study participants’ responses.

**Table 5:**
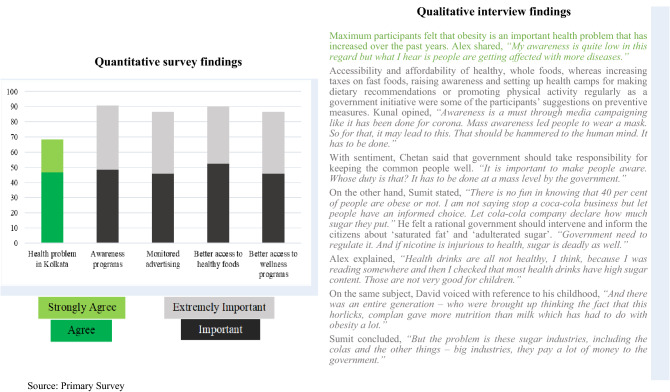
Juxtaposed findings of quantitative and qualitative investigation on preventing obesity.

## Discussion

### Environmental determinants

Participants in this study had reported encountering serious challenges posed by the built environment—most notably, the food environment. Participants shared their concerns about packaged foods and food advertisements, especially those of western origin, which have become the badge of belonging to a higher social status. Participants also shared their concerns about the affordability of healthy and organic food. They hinted at the profit-making food industry and the profit-making fitness industry. On the other hand, they found no discussions about food knowledge or awareness programs related to good health practices. Participants indicated that the food, medical, and weight loss industries were profiting from people's weaknesses. On the contrary, most participants felt they lived in a walkable, convenient, and safe neighbourhood. Nevertheless, the interview participants found the city's weather their perceived barrier to engaging in physical activities.

The relationship between environment and obesity is multifaceted and complex, involving communities, workplaces, and homes^6^. Prior research had established the link between obesity and the built environment^28^, as also found in our study. Obesogenic environments comprising food affordability, availability, marketing, etc., have been confirmed to encourage obesity in populations^5^. According to Jones and Bentham^29^, the modern diet includes high-energy–density foods as well as large portion sizes. When coupled with reduced physical activity, the energy balance gets tipped in favour of obesity. On the other hand, individual food preferences are often influenced by people’s culture^29^. The increasing income and literacy alongside urbanisation, improved infrastructure, and connection to the global trend are reshaping consumer preference and their demands in India^30^. Recent dependency on imported foods has simplified people's diets in an unhealthy manner, displacing the genetically diverse nutrient-rich whole foods in favour of more processed foods due to corporate branding^31^. Changes in food behaviour, such as the dependency on instant foods or the conventional diet, have roots in economic and social trends and self-choice. Consistent with the study's findings, numerous food and beverage advertisements and promotion appears on the internet, in magazines, in newspapers, and on television, with consequences that the general population may be ignorant of^6^ and can be challenging to overcome. Advertisements with food images primarily promote high-calorie, less nutritious foods and tend to encourage consumption, creating a poor food environment^6,7^. Harris et al.^32^ observed that most adults did not foresee the impact of food advertising on their eating behaviours. To sum up, the contemporary built environment encourages sedentary behaviours and the intake of more calories than required^6^. Hence, people now also tend to gain weight since they live in an obesogenic environment^7^.

Besides, participants also shared their concerns about food adulteration. Adulterating food products could cause adverse effects on human physiology, and it is plausible that it plays a significant role in increasing the rate of obesity in Kolkata. Even though no literature had been found that showed a link between food adulteration and obesity, a study on food contamination was not published and was conspired into silence^33^. The Annual Public Laboratory Testing Report by the Food Safety and Standards Authority of India (FSSAI), 2014–15, revealed that nearly one-fifth of the food items were misbranded or adulterated^34^. A study by Marshall et al. published in 2003 tested common vegetables (spinach, cauliflower, okra) that were found in the daily Indian diet in Delhi between May 2001 and June 2003 and found that 72% of the 222 spinach samples exceeded the maximum concentration of a pesticide residue as per the Indian standard^35^. In India, pesticide contamination of food is a big issue, especially in milk, vegetable and fruit. Despite that, government agencies in^33^ do not regularly test heavy metals in vegetables. These food safety incidents are caused mainly by lax regulations and poor standards^35^.

Policymakers and public health professionals should consider these factors while planning intervention programs. As the national income rise in developing nations like India, changes in dietary pattern and activity levels increase the risk of overnutrition while undernutrition is still there^7^. Since the urban rates of obesity are much higher than their rural counterparts^15^, urban residents should get access to a clean, healthy food environment.

### Health complications

Survey participants appeared to be well perceptive on physical health complications of obesity except for cancer. The interview participants frequently mentioned that obesity could increase the risk of developing diabetes, hypertension, cholesterol and heart diseases. They also linked female reproductive problems, haemoglobin issues, breathing troubles, thyroid, fatigue, uric acid, depression, gastric issues, arthritis, asthma, body pain and joint pain, kidney problems, migraine and carpal tunnel syndrome with obesity. However, few also asserted that some of these health complications might also be related to genetics^9^ and that one can be equally functional and have excess weight. On the contrary, the interviewees did not mention cancer, liver problems, sleep apnea or premature death as the physical health complications of obesity.

Participants manifested their understanding of several health complications of obesity, consistent with the findings of prior studies^36–38^. Obesity has been associated with several comorbidities and may lead to heart disease, high blood pressure, diabetes and other complications^9^. Besides, in another previous study, it was given that people of different bodyweight might have varying health outcomes due to the complex range of factors^37^.

Around 68.33 per cent (n = 82) of the survey participants said they had at least one health complication, while 17.5 per cent (n = 21) had three or more health complications. Altogether, these complications were more commonly found among female survey participants, with a BMI of 30 or higher and waist circumference of 102 cm or higher. On the other hand, 11 interview participants, three females and six males had at least one health complication, while two female participants had at least three or more complications with a BMI of 30 or higher and a waist circumference of 102 cm or higher. That suggests that higher BMI and waist circumference will likely elevate the risk of health complications. The four most-cited complications present among the participants were hypertension, thyroid, cholesterol and high blood glucose. These health complications and BMI tend to increase with higher age. In addition, a few interview participants also pinpointed concerns regarding the lack of health and nutrition education.

The associated physical health complications were also mentioned in the literature^7,9,38^. Chronic diseases like hypertension, obesity, and diabetes tend to emerge in the middle ages due to prolonged exposure to unhealthy lifestyles, specifically poor diet, lack of physical activity, and tobacco use^39^.

Among survey participants, fewer people agreed to have problems with walking, climbing stairs, doing household chores, and being depressed, whereas more agreed to have difficulty squatting and snoring. Among them, 37.5 per cent (n = 45) had three or more concerns, and only 2.5 per cent (n = 3) had all six. While the interview participants now and then mentioned having problems with walking, climbing stairs, doing household chores, and squatting and that they were depressed and snored. These concerns were also more commonly found in females, with a BMI of 30 or higher and a waist circumference of 102 cm or higher. It is known that snoring could be a symptom of sleep apnea^40^. Although 51.38 per cent of snoring survey participants did not report having sleep apnea, it is likely that more than 21.67 per cent of individuals had sleep apnea but were unaware of it.

Agrawal et al.^41^ also reported that many women had problems with walking, climbing stairs, squatting, and doing household chores because of their body weight. In addition, prior research^7,9^ also showed that individuals with excess weight were more likely to be depressed.

### Preventing obesity

At the community level, obesity was seen as a critical health problem in Kolkata, with other diseases rising. In the present study, more than half of the interview participants (n = 10) stated that they had gained weight over the years. Participants suggested that there should be awareness programs and knowledge dissemination on nutrition, health, healthy behavioural habits, promotion and easy access to healthy food options and other wellness programs, and promotion of participation in physical activities at the institutional and community level. At the social/public policy level, there should be regulation on fast foods and sugary beverages. Nevertheless, participants hinted at the association between the big food industry, influential stakeholders, bureaucracy, and the government.

The increasing prevalence of chronic diseases like hypertension, diabetes, obesity, cancer etc., perhaps implies that people are not consuming the right kind of food that their bodies are designed to have^42^. Hence, public health messages need to opt for a balance to convey health risks targeting the disease ‘obesity’ and not an individual^35^. Most importantly, the key to healthy living should be moderation, balance and variety^43^. Furthermore, the general population should be protected from 'toxic' food industries, which have been thoroughly established in the literature^44^. The food industry’s motive for profit-making target people’s craving for sugar and claims that all calories are equal^45^ and that the association between diet and obesity is bad science^33^. Conversely, Spell^46^ recommended, “Counting calories alone doesn’t work”, and people also need to look into the quality of the calorie. However, these profit-driven food businesses are enshrouded by multinational stakeholders who receive backing from powerful lobbyists and are well aligned with the political system^44^, making them a challenging target.

## Conclusions

The present study assessed the perception, environmental determinants, and health complications of excess weight in Kolkata using a concurrent mixed methods approach. Some environmental factors promoting obesity were the lack of access to healthy, fresh foods at a fair price and a lack of health awareness programmes, advertisements, and weather conditions in Kolkata. Interview participants also extended their concerns about food adulteration and the food industry. Participants repeatedly confirmed that obesity could increase the risk of developing diabetes, hypertension, cholesterol and heart diseases, whereas they periodically disagreed with the association between cancer and obesity. Further, participants felt squatting was challenging for them. Hypertension was the most common existing health complication found among the study participants. Several survey participants agreed that sleep apnea was a health concern of obesity; few already had it; however, no interview participants cited it as a health concern. Participants felt most concerned about the food environment. Hence, they suggested raising awareness, promoting and making access to healthy food and wellness programs more accessible, and regulating fast foods and sugary beverages at institutional, community and social/public policy levels, respectively, to prevent obesity.

The finding illustrates a safe environment and social and public policies promoting good health practices are essential to make an intervention. Henceforth, emerging from the results and discussion, further statements can be made. It is imperative to raise consumer awareness about food safety risks. Rigorous inspection and continuous monitoring of food products must be carried out to ensure food safety. India needs to regulate its pesticide usage following the latest scientific findings to ensure food safety and safeguard public health^33^. Obesity is a complex chronic disease that can challenge primary care treatment, and in the absence of specialist obesity treatment in India^13^, several people would be unable to maintain a healthy body weight^5^. Thus, healthcare services need to improve^47^. After the Ayushman Bharat Program (ABP) was announced in 2018, it was asserted that the success and effectiveness of Health and Wellness Centres would depend on a rapid transition from the policy stage to implementation, increased and dedicated funding by the government, participation of the community and other stakeholders, focus on population health interventions & public health services, political will, effective use of information and communication technology, among other factors^48^. Even though ABP could assist India in achieving universal health coverage, it would be untenable on its own and need to be combined with other initiatives^18^. Besides, improved policies governing the sale, marketing, and advertising of junk and fast foods, as opposed to controlled pricing of healthy whole foods, can directly or indirectly encourage healthy eating. Improved policies and programmes addressing the social, environmental, and commercial roots of obesity are fundamental to legitimate support systems for healthier lives^5^. Obesity is a pandemic and one of the most significant public health problems in recent years. Yet the government in India does not classify obesity as a disease; even more, it is subsumed under the nutrition agenda. At the very least, India needs to reform its healthcare systems to address new developing realities to achieve universal health coverage, as pledged in the National Health Policy 2017^20^. Even though political investment extends to trans-fat and sugar tax, very little financial investment is given to obesity. Although numerous medical organisations have clinical guidelines and recommendations for obesity, their uptake is poor because these guidelines lack government support^13^. In addition, in 2003, when WHO urged governments to grow more vegetables and fruits and pushed toward healthy food subsidies and food education to combat obesity, the government of India chose to criticise the approach^33^. Unless we take action by educating, raising awareness and challenging, it will continue to rise and escalate even in the rural areas and, even worse, among individuals with lower socio-economic positions, which would undoubtedly put an economic burden on the public health expenditure and especially on the individual resources. Furthermore, future research should raise and address food safety and health concerns.

## Supplementary Information

Supplementary Information.

## Data Availability

The datasets generated and/or analysed during the current study are not publicly available since this study is based on a primary survey but are available from the corresponding author upon reasonable request. A supplementary Appendix file has been provided for the survey items.

## Abbreviations

ABP: Ayushman Bharat Program
BMI: Body Mass Index
FSSAI: Food Safety and Standards Authority of India
IERB: Institutional Ethics Review Board
JNU: Jawaharlal Nehru University
NFHS: National Family Health Survey
NIH: National Institute of Health
NCD: Non-communicable disease
OBC: Other Backward Classes
PCOS: Polycystic ovary syndrome
PI: Principal investigator
SC: Scheduled Caste
ST: Scheduled Tribe
WC: Waist circumference
WHO: World Health Organization
