# Supplementary material for: Perception, environmental determinants, and health complications of excess weight in India: a mixed methods approach
Source: Sci Rep. 2023 Apr 11;13:5868. doi: 10.1038/s41598-023-31016-w (PMC10088635; doi:10.1038/s41598-023-31016-w)
Supplement: Supplementary file 1 — Supplementary Information. [file 41598_2023_31016_MOESM1_ESM.docx]

**Appendix**

| **Serial No.** | **Survey Items** | **Responses** | | | | | **Missing** |
| --- | --- | --- | --- | --- | --- | --- | --- |
|  |  | **Strongly disagree** | **Disagree** | **Neither** | **Agree** | **Strongly agree** |  |
| 1 | Healthy food and fresh produce | 13 (11.11%) | 32 (27.35%) | 14 (11.97%) | 39 (33.33%) | 19 (16.24%) | 3 |
| 2 | Fast food | 4 (3.33%) | 4 (3.33%) | 1 (0.83%) | 47 (39.17%) | 64 (53.33%) |  |
| 3 | Food advertising | 10 (8.33%) | 26 (21.67%) | 17 (14.17%) | 44 (36.67%) | 23 (19.17%) |  |
| 4 | Coronary Artery Disease | 1 (0.83%) | 6 (5%) | 5 (4.17%) | 69 (57.5%) | 39 (32.5) |  |
| 5 | Hypertension | 1 (0.83%) | 3 (2.5%) | 11 (9.17%) | 57 (47.5%) | 48 (40%) |  |
| 6 | Cholesterol Problems | 1 (0.83%) | 4 (3.33%) | 4 (3.33%) | 62 (51.67%) | 49 (40.83%) |  |
| 7 | Type 2 Diabetes | 1 (0.84%) | 8 (6.72%) | 7 (5.88%) | 68 (57.14%) | 35 (29.41%) | 1 |
| 8 | Cancers | 12 (10%) | 39 (32.5%) | 37 (30.83%) | 24 (20%) | 8 (6.67%) |  |
| 9 | Liver Problems | 1 (0.83%) | 19 (15.83%) | 19 (15.83%) | 60 (50%) | 21 (17.5%) |  |
| 10 | Gastroesophageal Reflux Disease | 4 (3.33%) | 14 (11.67% | 12 (10%) | 72 (60%) | 18 (15%) |  |
| 11 | Sleep Apnea | 5 (4.17%) | 36 (30%) | 26 (21.67%) | 42 (35%) | 11 (9.17%) |  |
| 12 | Asthma | 4 (3.33%) | 23 (19.17%) | 20 (16.67%) | 52 (43.33%) | 21 (17.5%) |  |
| 13 | Arthritis | 1 (0.83%) | 7 (5.83%) | 9 (7.5%) | 62 (51.67%) | 41 (34.17%) |  |
| 14 | Premature death | 4 (3.33%) | 14 (11.67%) | 11 (9.17%) | 61 (50.83%) | 30 (25%) |  |
|  |  | **Yes** | **No** |  |  |  |  |
| 15 | Hypertension | 45 (37.5%) | 75 (62.5%) |  |  |  |  |
| 16 | High Blood Glucose | 20 (16.67%) | 100 (83.33%) |  |  |  |  |
| 17 | Sleep Apnea | 26 (21.67%) | 94 (78.33%) |  |  |  |  |
| 18 | Asthma | 13 (10.83%) | 107 (89.17%) |  |  |  |  |
| 19 | Cholesterol | 24 (20%) | 96 (80%) |  |  |  |  |
|  |  |  |  |  |  |  |  |
|  |  | **Strongly disagree** | **Disagree** | **Neither** | **Agree** | **Strongly agree** |  |
| 20 | Walking | 38 (31.67%) | 43 (35.83%) | 10 (8.33%) | 25 (20.83%) | 4 (3.33%) |  |
| 21 | Climbing | 30 (25%) | 37 (30.83%) | 9 (7.5%) | 36 (30%) | 8 (6.67%) |  |
| 22 | Household chores | 38 (31.67%) | 51 (42.5%) | 13 (10.83%) | 13 (10.83%) | 5 (4.17%) |  |
| 23 | Squatting | 22 (18.33%) | 31 (25.83%) | 9 (7.5%) | 48 (40%) | 10 (8.33%) |  |
| 24 | Snore | 15 (12.5%) | 16 (13.33%) | 19 (15.83%) | 54 (45%) | 16 (13.33%) |  |
| 25 | Depressed | 31 (25.83%) | 46 (38.33%) | 17 (14.17%) | 19 (15.83%) | 7 (5.83%) |  |
| 26 | Health awareness programs | 20 (17.09%) | 44 (37.61%) | 18 (15.38%) | 31 (26.5%) | 4 (3.42%) | 3 |
| 27 | Wellness, recreational facilities, Walkability | 4 (3.33%) | 17 (14.17%) | 11 (9.27%) | 68 (56.67%) | 20 (16.67%) |  |
| 28 | Climate | 8 (6.67%) | 29 (24.17%) | 16 (13.33%) | 53 (44.17%) | 14 (11.67%) |  |
| 29 | Garbage or litter | 28 (23.53%) | 43 (36.13%) | 15 (12.61%) | 26 (21.85%) | 7 (5.88%) | 1 |
| 30 | Stray dogs | 40 (33.61%) | 42 (35.29%) | 16 (13.45%) | 13 (10.92%) | 8 (6.72%) | 1 |
| 31 | Neighbourhood safety | 28 (23.73%) | 37 (31.36%) | 21 (17.8%) | 26 (22.03%) | 6 (5.08%) | 2 |
|  |  |  |  |  |  |  |  |
| 32 | Public transport | 24 (20%) | 56 (46.67%) | 21 (17.5%) | 16 (13.33%) | 3 (2.5%) |  |
| 33 | Health problem in Kolkata | 4 (3.33%) | 14 (11.67%) | 20 (16.67%) | 56 (46.67%) | 26 (21.67%) |  |
|  |  | **Not at all important** | **Slightly important** | **Fairly important** | **Important** | **Extremely important** |  |
| 34 | Educational programs | 1 (0.83%) | 3 (2.5%) | 7 (5.83%) | 58 (48.33%) | 51 (42.5%) |  |
| 35 | Monitored advertising | 3 (2.5%) | 3 (2.5%) | 10 (8.33%) | 55 (45.33%) | 49 (40.83%) |  |
| 36 | Nutritious food options | | 5 (4.17%) | 7 (5.83%) | 63 (52.5%) | 45 (37.5%) |  |
| 37 | Wellness programs | 2 (1.67%) | 3 (2.5%) | 11 (9.17%) | 55 (45.83%) | 49 (40.83%) |  |
